# Supplementary material for: Identification of a Novel Mutation Exacerbated the PSI Photoinhibition in pgr5/pgrl1 Mutants; Caution for Overestimation of the Phenotypes in Arabidopsis pgr5-1 Mutant
Source: Cells. 2021 Oct 26;10(11):2884. doi: 10.3390/cells10112884 (PMC8616342; doi:10.3390/cells10112884)
Supplement: Supplementary file 1 [file cells-10-02884-s001.zip › cells-1396360 -SM figures .pdf]

cells MDPI

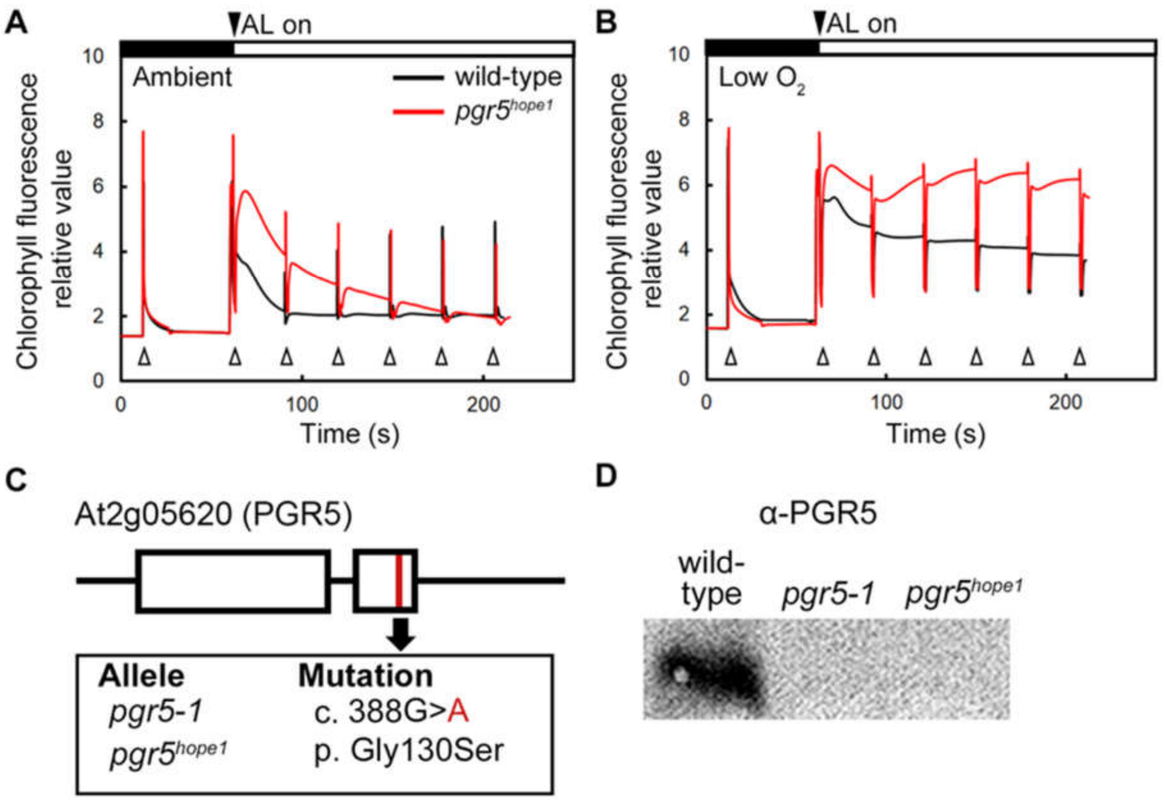

Supplementary Figure S1: *pgr5*^{hope1} screening method and mutation identification.

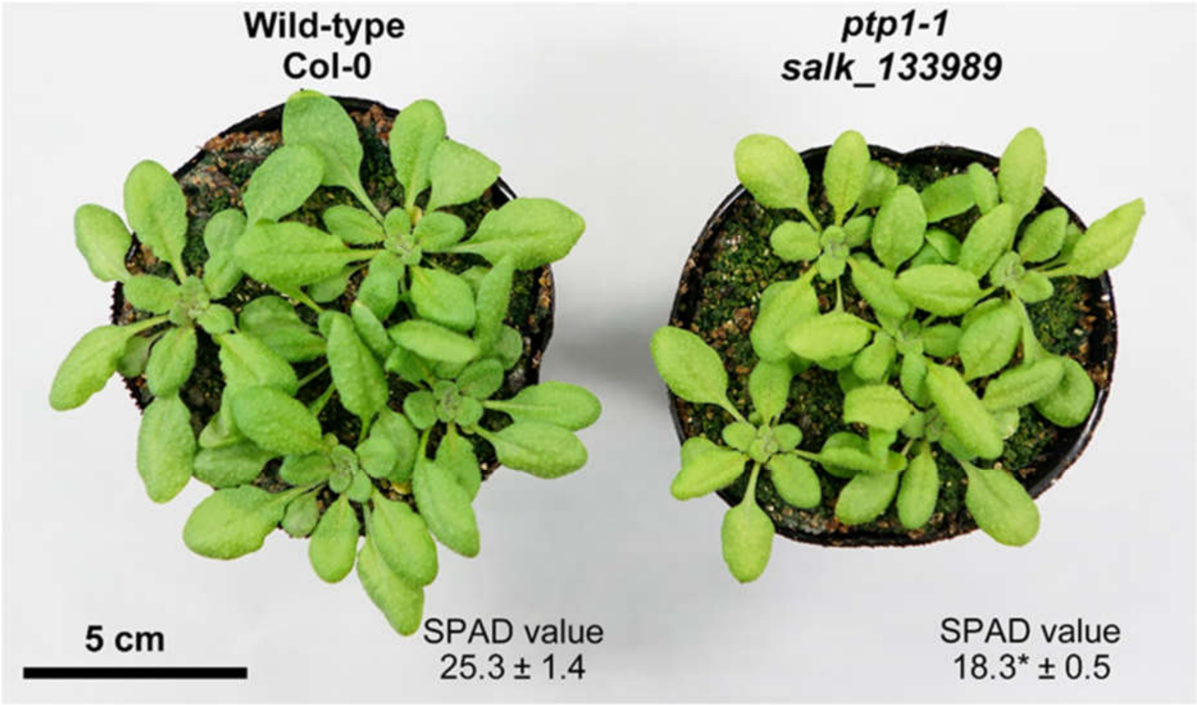

Supplementary Figure S2: The growth phenotype of *ptp1-1* (*salk_133989*) mutant.

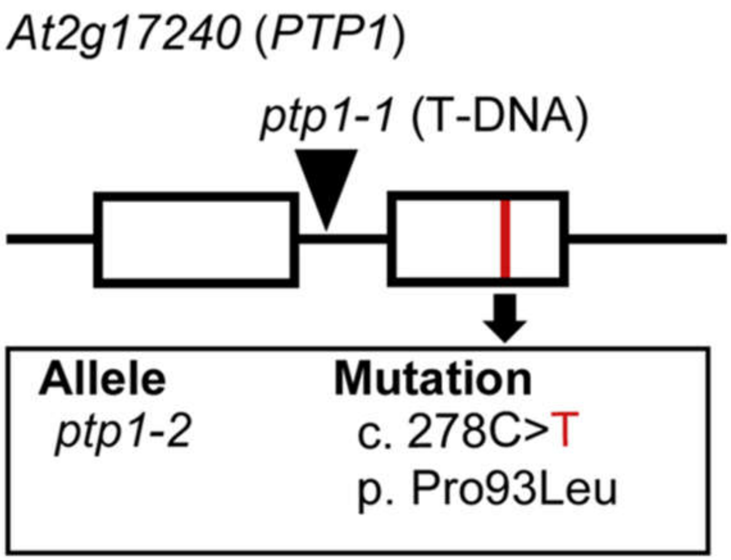

Supplementary Figure S3: The *PTP1* mutations in *ptp1-1* and *pgr5-1*.

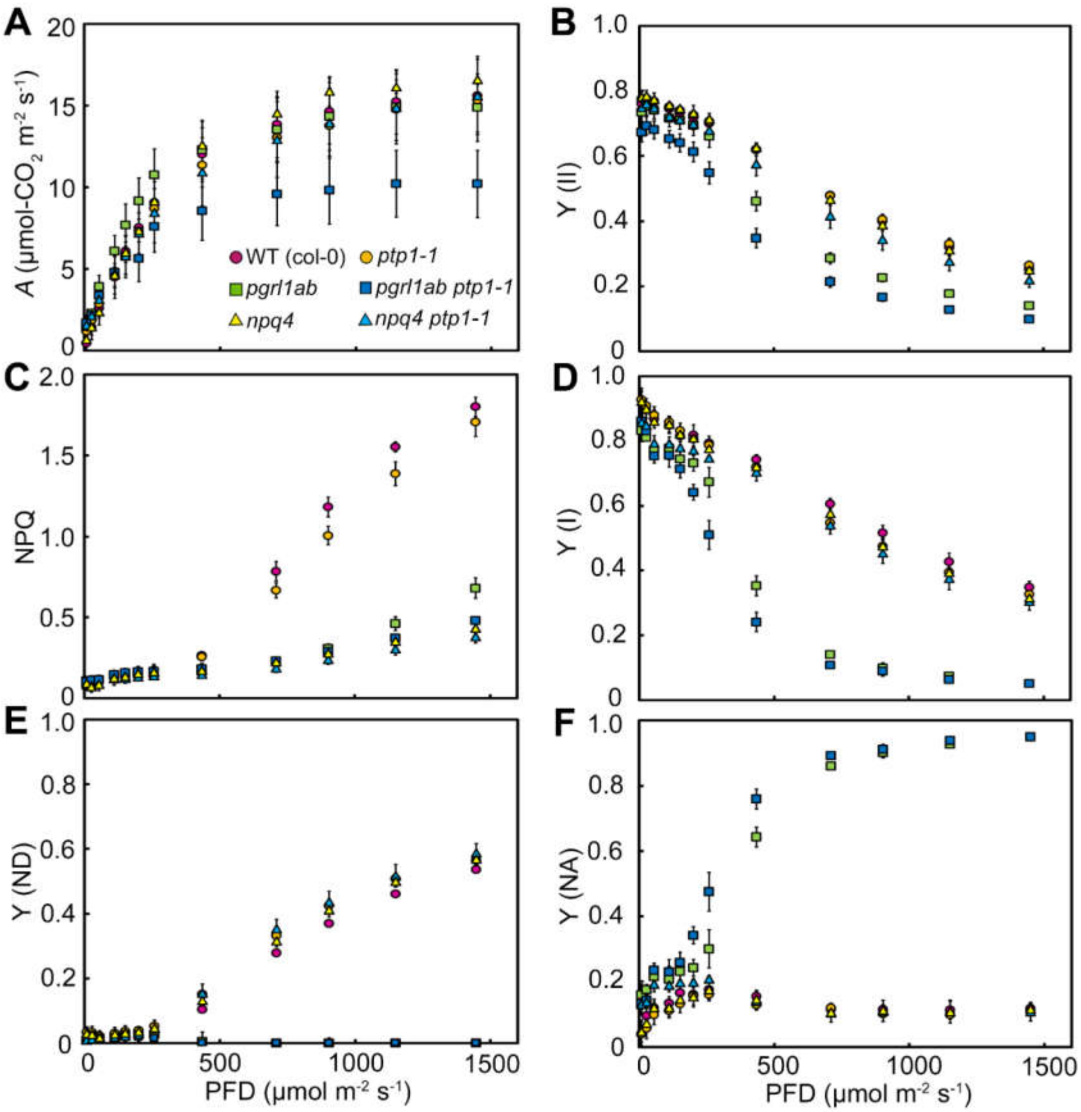

.

Supplementary Figure S4: The photosynthesis capacity of *pgrl1ab* and *pgrl1ab ptp1* mutants.

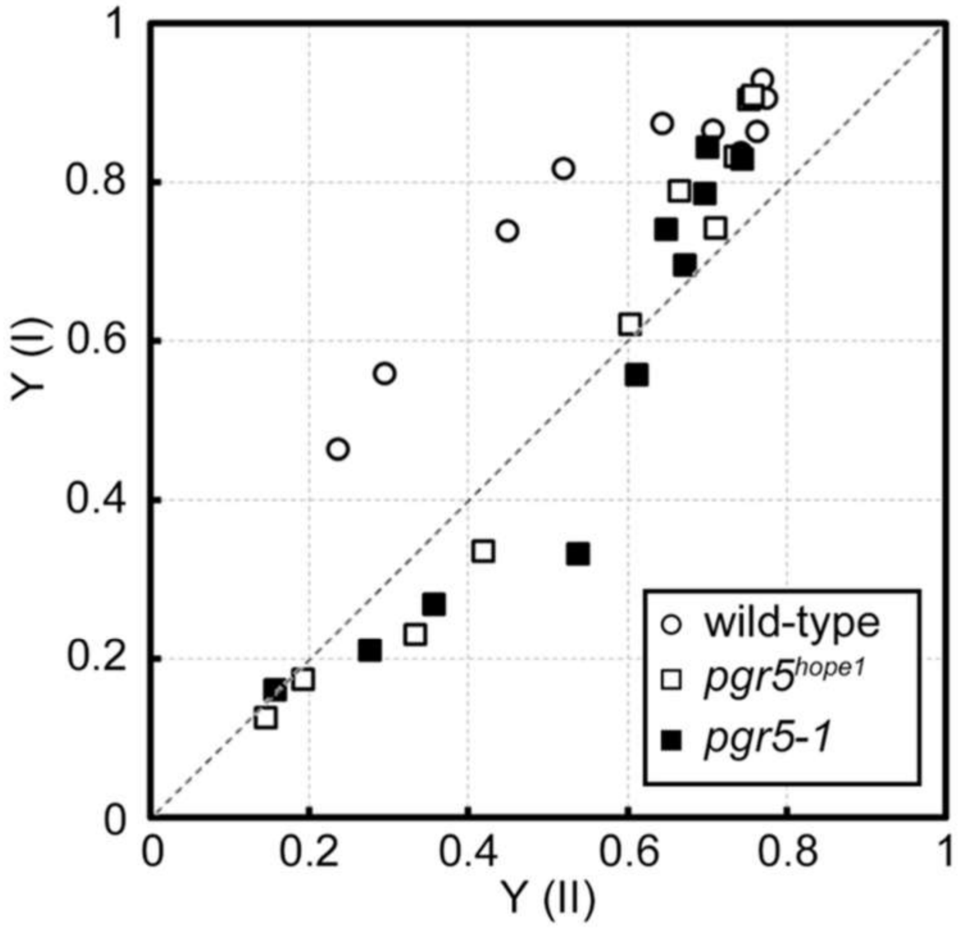

Supplementary Figure S5: The extent of photoinhibition in PSII and PSI by constant intense light using *pgrl1ab* and *pgrl1ab ptp1* mutants.

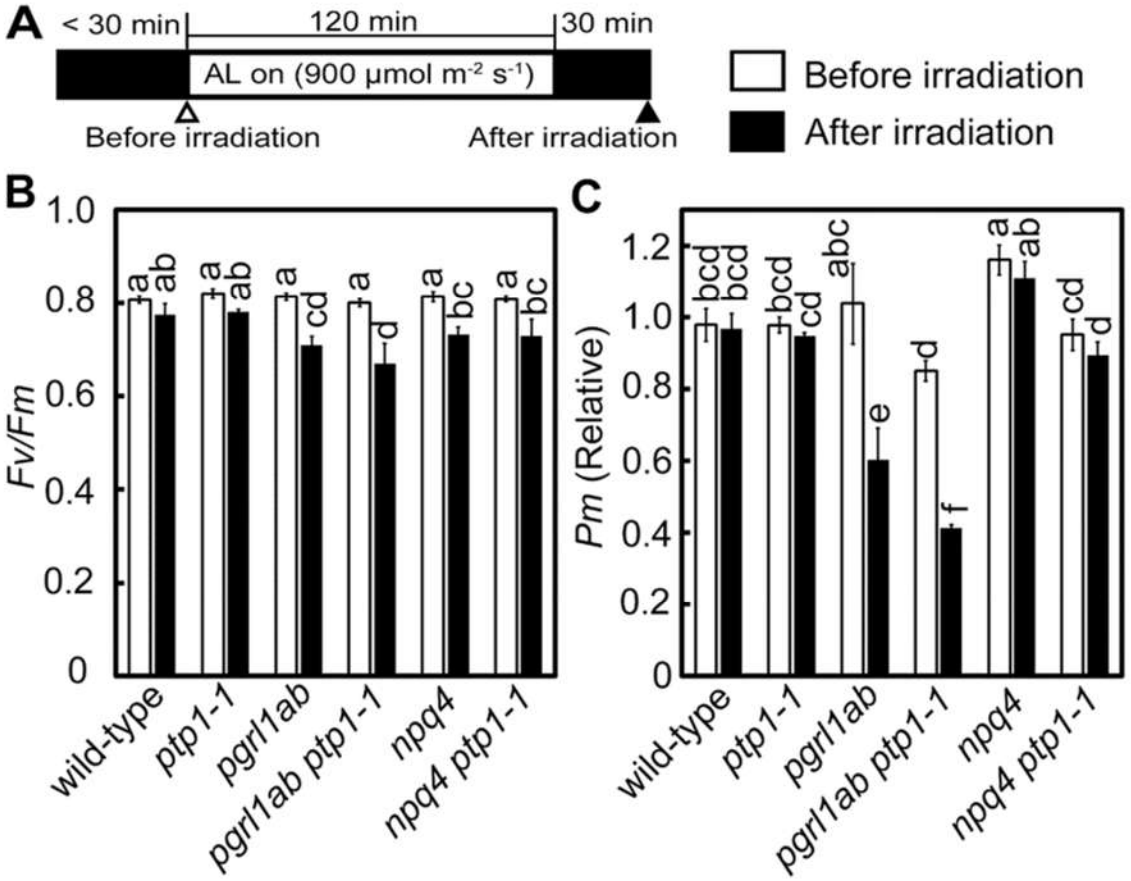

Supplementary Figure S6: The relationship between Y(II) and Y(I) in wild-type, *pgr5*^{hope1} and *pgr5-1*.

Supplementary Table S2: Primer information used in this study.

Primer	Primer sequence	
SALK_133989_LP	GAAGGTTCGAATTTCGAAAGG	Specific primers for selection of *SALK_133989* mutant
SALK_133989_RP	ACTATCAAAACGCAAACGCAG	
Forward primer for pgr5 cloning	CACCATGGCTGCTGCTTCGATTTCTG	Specific primers for pgr5 cDNA cloning and insertion into D-TOPO entry clone
Reverse primer for pgr5 cloning	CTAAGCAAGGAAACCAAGCCTC	
Forward primer for ptp1 cloning	CACCATGGCGTCGCTTTGCTCTGC	Specific primers for ptp1 cDNA cloning and insertion into D-TOPO entry clone
Reverse primer for ptp1 cloning	CTATTGCTGCTTCTCGGGTTGTTC	

Supplementary Table S2: The relative amount of PGR5 protein in each mutant (per chlorophyll) The PGR5 protein was relatively quantified based on the results of immunoblotting. Each number shows the relative value when the wild-type (Col-0) is 1.0. Values are means ± SD. (*n* = 3) n.d. indicates not detectable.

genotype	Relative amount of PGR5 protein per chlorophyll
**Wild-type**	
Col-0	1.0 ± 0.2
gl-1	1.0 ± 0.2
**mutants**	
pgr5-1 (gl-1)	n.d.
pgr5 hope1 (gl-1)	n.d.
ptp1-1 (Col-0)	1.4 ± 0.2
pgr5-1 PGR5	2.9 ± 0.6
pgr5 hope1 PGR5	2.7 ± 0.5
pgrl1ab (Col-0)	n.d.
pgrl1ab ptp1-1 (Col-0)	n.d.

.
